# Open questions in understanding life’s origins

**DOI:** 10.1038/s42004-021-00448-8

**Published:** 2021-02-02

**Authors:** Christopher J. Butch, Markus Meringer, Jean-Sebastien Gagnon, H. James Cleaves

**Affiliations:** 1 Department of Biomedical Engineering, College of Engineering and Applied Sciences, Nanjing University, Nanjing, China; 2 Earth-Life Science Institute, Tokyo Institute of Technology, Tokyo, Japan; 3 Blue Marble Space Institute for Science, Seattle, WA USA; 4 German Aerospace Center (DLR), Earth Observation Center (EOC), Oberpfaffenhofen-Wessling, Germany; 5 Physics Department, Norwich University, Northfield, VT USA; 6 Department of Earth and Planetary Sciences, Harvard University, Cambridge, MA USA; 7 Institute for Advanced Study, Princeton, NJ USA; 8 Center for Chemical Evolution, Georgia Institute of Technology, Atlanta, GA USA

**Keywords:** Computational chemistry, Origin of life

## Abstract

The chemical space of prebiotic chemistry is extremely large, while extant biochemistry uses only a few thousand interconnected molecules. Here we discuss how the connection between these two regimes can be investigated, and explore major outstanding questions in the origin of life.

As we search for habitable and inhabited planets beyond Earth, defining life and understanding how it originates is critical to designing life detection missions^1^. Though scientists from many fields have tried to understand the origins of life, and many hypotheses exist, a precise definition of life remains elusive^2^, and we do not presently know how life began.

From interstellar observations and carbonaceous meteorites, it is known that complex organic chemistry occurs widely in primitive solar system environments (e.g., ref. ^3^). Conversely, we have the single data point of the chemistry produced by our biosphere. The space between these data points is sparsely filled by experiment, model, and hypothesis. Experimentally addressing the chemical origins of life is complicated by the size of organic chemical space^4^, and the tandem sparsity and complexity of reactions which could give rise to autocatalytic, replicative and ultimately living chemistry. A large amount of chemistry remains to be explored, and it is likely the field will benefit from a combination of experimental, observational and computational studies. For example, computational chemists can algorithmically explore chemical space using graph “grammars”^5^ much more rapidly than “wet” chemists can experimentally, though such computations are still hampered by accuracy and computational capacity^6^.

Origins of life models, regardless of biases along heterotrophic/autotrophic axes^7^, all depend on the origin of chemical reaction *networks*. But life is more than a collection of reactions and compounds, it is a *systemic* phenomenon characterized by feedbacks that modulate kinetics. Within reaction networks, slight differences in reactivity can cause large systemic effects. *Network closure*, in which the edges (in this case reactions) and nodes (here, chemical compounds) of a network form a single connected component^8^, is a unifying concept defining hierarchically functional and selectable biological units (e.g., metabolic pathways, genes, organelles, cells, species, ecosystems, etc.^9,10^). Network development may also lead to the emergence of novel phenomena^9^: new graph rewriting rules may be created by networks, for example by creating new phases which alter some reactions’ kinetics. How networks achieve closure and simultaneously bring about “internal causation,” in which species are created by catalytically closed reaction networks, is a complex but addressable question. Previous models have demonstrated how these types of emergent systemic properties may have contributed to the origins of life^11–13^, but models with more precise chemical predictivity are needed.

There are several fundamental problems in understanding the chemical origins of life which require study in the context of networks and their closure (summarized in Fig. 1).

1. *Understanding how individual chemical reactions concatenate to expand reaction networks*. To understand the transition from prebiotic chemistry to biochemistry, it is important to first understand the generation of complex chemical networks from some kind of “primordial” feedstock. “Wet” chemists approach this problem by doing experiments, and defining rules to predict outcomes of reactions based on these experiments. This approach is of course the most realistic, but studying the whole chemical possibility space could be time consuming (if at all possible). A more time efficient approach is to formalize chemical rules using “graph grammars“^5,6^, and use those rules to predict “real-world” chemistry in silico. However, this type of approach leaves many problems unresolved, for example important reaction pathways may be excluded because they are not intuited using human or machine-learned screening. The expansion of networks using formalized grammars creates complex reaction networks, but kinetics affect the abundances of products, which influence downstream network dynamics. Reaction networks primarily grow and self-limit when they run out of feedstocks, not because they are limited by “possibility space”.
2. *Exploring the relationship between networks and chemically realistic catalysis*. As reaction networks grow, they may create new compounds capable of influencing network development by acting as catalysts, which enable new reactions or enhance one or more reactions relative to the network. The creation of network-influencing catalysts by reaction networks has been addressed by Kauffman’s binary polymer model, which examines how catalysts endowed with randomly-assigned kinetic enhancement properties affect network closure^12^. Diversity-generating reactions might not be open-ended because implementation of all possible reaction rules on all generated substrates leads to no new implementable reactions or products. Some reaction networks may never grow or change their explorable properties because they generate no new phenomena (e.g. catalysts or phases) or reactions, while others become truly open-ended.
  Unlike in the Kauffman model, however, real catalysis is an inherently three dimensional problem wherein the catalytic molecule interacts with a reactant or transition state to alter the energetics of the reaction. Because of this three dimensional nature, it is then reasonable to assume that catalysis may be transferable to other reactions with similar shape and electrotopological character. It is presently unknown how this type of three dimensional biasing effects the behavior of reaction networks. Rule-based reaction network expansion does not have an inbuilt mechanism for the discovery of this type of catalysis, or the estimation of its kinetic effects. Reaction-expansion methods presently poorly predict these kinds of reaction feedbacks, including ones that may steer stereochemistry.
3. *Understanding the principles of spontaneous phase separation leading to cellularization*. Diversity-generating reaction networks may create products capable of producing *phase separation*, which provides new playgrounds for network growth, including covalent and non-covalent aggregation and compartmentalization, which forms the basis of phenomena like Polymerization-Induced Self Assembly^14^. The types of phases prebiotic chemistry is capable of generating, and the nature of their interactions, are likely more complex than generally appreciated^15^, and new phases may in turn create novel network dynamics^6^.
4. *Understanding the origins of activation and group transfer processes*. Biochemistry enables formally thermodynamically impossible reactions to occur by coupling activation to environmental energy sources and novel catalytic processes. Network generation assumes reactions proceed in thermodynamically favored directions, thus they must be fed. How chemical energy was supplied to primitive environments would have affected how prebiotic reaction networks developed. Before chemical networks were able to produce and take advantage of the multiple benefits created by the emergent phenomena discussed above, it is unclear how networks adapted to the changing availability of chemical energy provided by the environment. There was likely a temporal and qualitative order to the development of energy exploitation processes. Using models employing realistic thermodynamics, England^16^ has suggested how external energy inputs can drive systems towards increased local order, while increasing entropy generation globally.
5. *Understanding the origins of hierarchically structurally decoupled catalytic encoding*. The central dogma describes how contemporary biochemical information flows essentially unidirectionally between genotype and phenotype occurs from DNA to RNA to protein in cells^17^, providing a connection between genetic inheritance, mutation and natural selection. How this mapping arose is perhaps the largest open question in the emergence of life. This information flow is mediated by sophisticated covalent and non-covalent interactions and belies the possibility that there may have been alternative earlier flows during the early chemical and biochemical evolution.

**Fig. 1:**
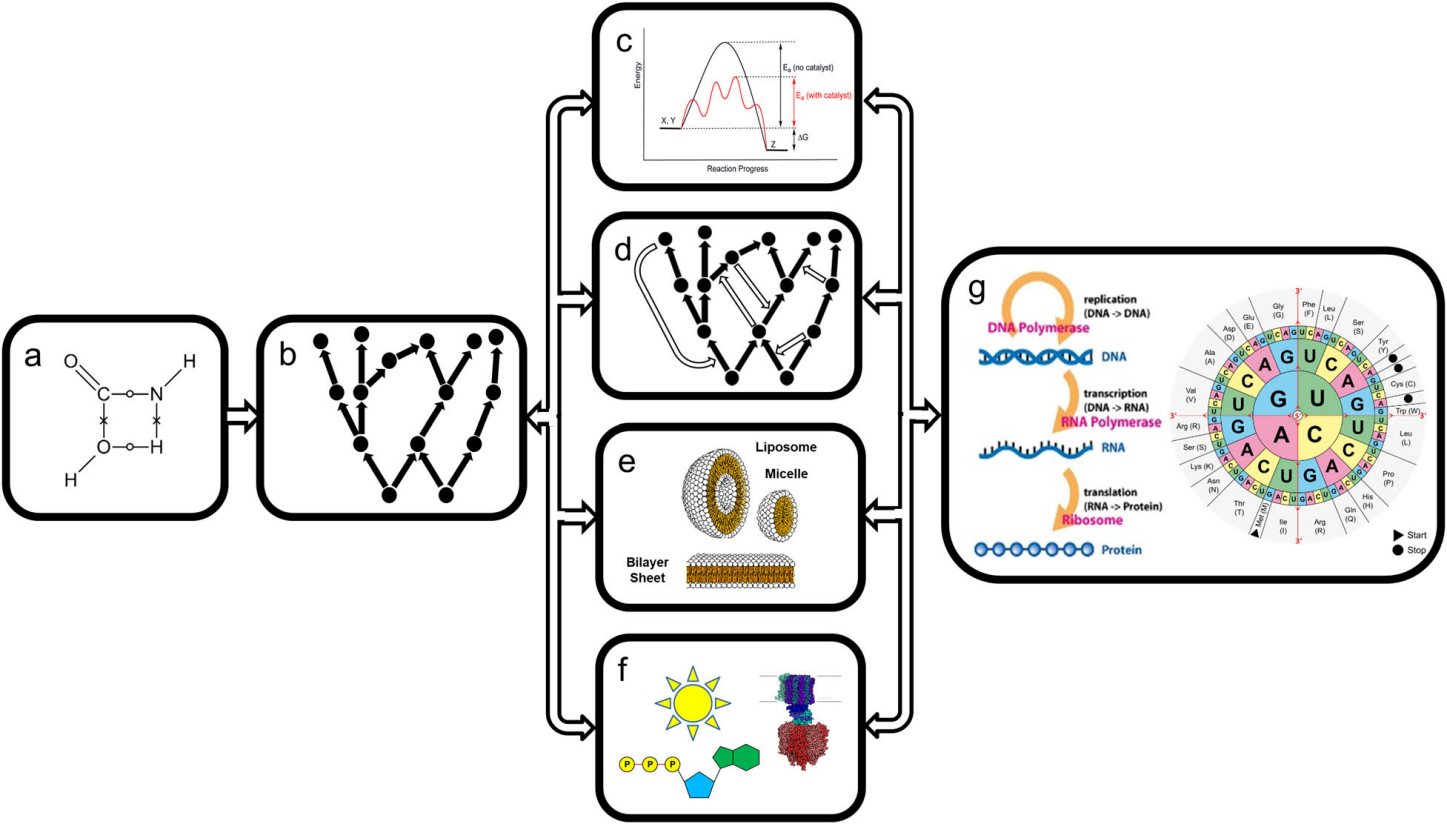
Workflow for studies of the chemistry of life’s origins. Prebiotic reactions proceed according to mechanisms defined by physical organic chemistry (**a**) to make complex product suites (**b**), as determined by kinetics and thermodynamics. Products of these simple networks may change the rules and restrictions of their generation causing the network to become more self-directed and life-like. Products may act as catalysts which lower energy barriers and enable the discovery of new chemistry (**c**) or steer the flux of compounds through certain preferred pathways which can become mutually reinforcing, leading to network closure (**d**). Products may also contribute to phase separation and another form of closure, encapsulation (**e**). Emergence of still more complex phenomena, such as activating chemistries and cellular capacitors, likely depend on multiple layers of network feedbacks (**f**). Finally the emergence of language-like processes may occur in a network with sufficient control to decouple structure and function (**g**). Feedbacks giving rise to such processes are presently hard to predict. Images are sourced as follows: Panel c: public domain licensing (https://commons.wikimedia.org/wiki/File:CatalysisScheme.png); Panel **e**: public domain licensing (https://commons.wikimedia.org/wiki/File:Phospholipids_aqueous_solution_structures.svg); Panel **f**: redrawn from Creative Commons Attribution-Share Alike 3.0 Unported licensing (https://commons.wikimedia.org/wiki/File:Atp_synthase.PNG); Panel **g**: Creative Commons Attribution-Share Alike 3.0 Unported licensing (https://commons.wikimedia.org/wiki/File:Aminoacids_table.svg).

Language is one of the “breakout” phenomena which define humanity^18^. A language is a codified system of representation in which objects are represented as symbols. “Grammars” are basic rules which allow for “syntaxes” (concatenated rules which make sense within a language’s rules) at each level of representation to function. The connection between lower-level precisely-defined rules and more abstract language rules means that low-level rules interactively and stochastically construct “meaning” in languages. Chemically, this allows for indirect coupling of structure-based catalysis with larger reflex arcs. Viewed in this light, the phenomenon of hierarchical emergence is reminiscent of the analogical mapping between the genetic code and human language.

Language involves the mental encoding of concept, followed by expression via speech, followed by auditory or visual reception, and finally mental decoding into received “meaning.” Molecular interactions may not be directly “about” anything, molecules react and interact according to rules. The concept of “something being about something else” depends on concatenated rules and systemic context^19^, and is a form of *meta-catalysis*.

## Outlook

Undoubtedly, understanding these interlinked and emergent phenomena will benefit from close collaboration between experimental and computational chemists. For example, discovering small molecule catalysts requires algorithms which can evaluate potential intermolecular interactions and their effects on network kinetics. Literature-based prediction is currently only poorly capable of predicting the effects of such catalysis, however medicinal chemists routinely use docking techniques to rapidly screen non-covalent interactions of drug candidates with enzymes^20^. These methods may be adaptable to rapid screening for potential catalytic interactions among reaction network products expanded using graph transformation rules.

At the level of understanding phase separation and self-assembly, the molecular properties (e.g., K_ow_, LogP, LogD, etc.) that enable non-covalent molecular aggregation can be estimated using chemoinformatics techniques^21^. However, these methods are presently imprecise since the formation of micelles or vesicles depends on understanding higher order solvent-interaction effects, requiring the use of still other computational techniques. Such effects require their own parameterization and evaluation, but it should be possible to cull the local regions of network chemistry which can give rise to them to save computation time, and careful in vitro screening of large amphiphile libraries would improve the predictivity of computational methods.

Rapid methods for the discovery of *meta-catalysis* may provide insight into the observed “jumps” evident in evolution^18^. Meta-catalysis is *indirect*, and may help explain the origins of heritable and mutable information coding, which enable the responsiveness and adaptivity of reaction networks to external stimuli. To discover meta-catalytic phenomena, in silico generative networks need to be analyzed using computationally intensive tests at the level of interactional catalysis, which is not presently simple, and then re-evaluated with every other molecule in a network, to see if new “meaningful” hypergraphs are created. The models in turn need careful vetting using wet chemistry.

The RNA World concept provides an example of what is lacking in origins models. Chemists have provided ever more “prebiotically plausible” syntheses of RNA^22^, and SELEX experiments have shown it is easy to isolate and amplify molecules which bind target molecules^23^, but a recent computational exploration of nucleic acid space found more than a million possible alternative backbones to deoxyribose/ribose life as we know it uses^24^. None of these approaches can presently address how reaction networks make compounds in thermodynamically and kinetically feasible ways, and predict how the resulting products interact to modify the reaction networks which produce them.

## Summary

The transition from reaction mechanisms predetermined by physical chemistry to catalyzed network-pruning reactions, to self-sorting phase generating reactions and indirect catalysis form fundamental questions on the origin of life. Both experimental and computational approaches will help understand these transitions. However, numerous problems need to be solved to be able to apply computation in meaningful, tractable ways. Borrowing and adapting techniques from other disciplines is likely the most straightforward method of making progress in this area. Refining these approaches will help focus studies in experimentally testable ways.
